# Fine Particulate Matter Related to Multiple Sclerosis Relapse in Young Patients

**DOI:** 10.3389/fneur.2021.651084

**Published:** 2021-05-21

**Authors:** Edouard Januel, Boris Dessimond, Augustin Colette, Isabella Annesi-Maesano, Bruno Stankoff

**Affiliations:** ^1^Assistance Publique des Hôpitaux de Paris, APHP, Hôpital Saint Antoine, Neurology Department, Paris, France; ^2^Sorbonne Université and INSERM, Épidémiologie des maladies Allergiques et Respiratoires, Institut Pierre Louis d'Epidémiologie et Santé Publique, Paris, France; ^3^Atmospheric Modelling and Environmental Mapping Unit, INERIS, BP2, Verneuil-en-Halatte, France; ^4^Sorbonne Universités, Brain and Spine Institute, ICM, Hôpital de la Pitié Salpêtrière, Inserm UMR-S 1127, CNRS UMR 7225, Paris, France

**Keywords:** multiple sclerosis, relapse, air pollution, particulate matter 2.5 μm, young

## Abstract

**Objective:** Particulate matter (PM) of aerodynamic diameter smaller than 10 μm (PM_10_) has been associated with multiple sclerosis (MS) relapse. However, the impact of smaller PM with a greater ability to penetrate human organism has never been assessed. We evaluated the impact of PM smaller than 2.5 μm (PM_2.5_) on the risk of MS relapse.

**Material and Methods:** In a case-crossover study, we included 2,109 consecutive hospitalizations likely due to MS relapse in day hospital in 5 MS centers in the Paris area from January 2009 to December 2013. For each hospitalization, the natural logarithm of the average weekly PM_2.5_ concentrations (μg/m^3^) at the patient's residence address during each of the 6 weeks (week[0] to week[−5]) preceding admission was compared with the concentration during the previous week, using a conditional logistic regression adjusted on temperature, flu-like syndrome rate, pollen count, and holiday period.

**Results:** PM_2.5_ average concentration during week[−3] was significantly associated with the risk of hospitalization for MS relapse [OR = 1.21 (CI 1.01;1.46)]. The association was stronger in patients younger than 30 years [OR=1.77 (CI 1.10; 2.83)].

**Conclusion:** Our study demonstrates an association between exposure to PM_2.5_ and MS relapse, particularly in young people.

## Introduction

Ambient air pollution, and especially particulate matter (PM), has emerged as a global major health concern, leading to the death of 8.9 million people worldwide (1).

PM defined as any gaseous or solid particle suspended in the air is subdivided according to size: respirable (PM_10_, diameter <10 μm), fine (PM_2.5_, diameter <2.5 μm), and ultrafine (PM_0.1_, diameter <0.1 μm). PM_10_ and PM_2.5_ often derive from different emissions sources and also have different chemical compositions. Emissions from combustion of gasoline, oil, diesel fuel, or wood produce much of the PM_2.5_ pollution found in outdoor air, as well as a significant proportion of PM_10_. PM_10_ also includes dust from construction sites, landfills, and agriculture, wildfires and brush/waste burning, industrial sources, wind-blown dust from open lands, pollen, and fragments of bacteria (2). The various PMs also differ regarding their potential to penetrate the human body, as both PM_2.5_ and PM_0.1_ can reach the plasmatic circulation, whereas PM_0.1_ may also be able to reach the CNS through the blood-brain barrier (BBB) (3, 4) and the olfactory bulb (5).

PM_2.5_ exposure was suggested to be associated with the onset of Alzheimer disease (6), stroke (7), amyotrophic lateral sclerosis (8), and MS (9, 10). Several mechanisms have been evoked to explain this relationship: systemic and cerebral inflammation, oxidative stress, and breakdown of the blood-brain barrier (11). PM_10_ has also been suggested as a risk factor for MS relapse (12–14) and new lesion formation (15), but the effect of PM_2.5_ exposure on MS relapse risk has never been explored. However, PM_2.5_ composition differs from PM_10_ and can penetrate deeper in the lung, therefore inducing a stronger alveolar and systemic inflammation (16). We conducted here a study to determine the specific impact of PM_2.5_ on MS relapse. As MS frequently starts in young adults with more inflammatory phenotypes in younger subjects, we then selectively investigated whether the relation between PM_2.5_ and MS relapse depended on age.

## Methods

### Definition of Hospitalizations Related to MS Relapses

A case-crossover design has been chosen to investigate the association between PM_2.5_ concentrations and MS relapses, thus making every patient his/her own control, at different periods of exposure. The study area was Ile de France, comprising the city of Paris and surrounding areas, for a total of around 12 million inhabitants.

Hospitalizations were identified using the French administrative database called “Programme de Médicalisation des Sytèmes d'Information (PMSI)” in which the main diagnosis of every hospitalization is referenced. For the purpose of the present study, inclusion criteria targeted hospitalizations of patients whose personal address was located in Ile de France, with a day-hospital stay of 3 to 6 days, with principal hospitalization diagnosis of MS (G35) or non-carcinologic chemotherapy associated with MS (Z512, related diagnosis G35), from January 1, 2009 to December 31, 2013. During this period, relapse treatment by methylprednisolone was only administered IV and mostly through serial day-hospital for 3 to 5 days.

As no specific diagnosis code exists for MS relapse, we built a temporal algorithm in order to exclude hospitalizations for patients with MS without relapse. Criteria used in the algorithm were as follows: (1) MS hospitalizations separated by <7 days were considered as the same hospitalization; (2) for each patient, if two MS hospitalizations were separated by more than 7 days but <75 days, these hospitalizations were considered as repeated hospitalizations; (3) if a patient had three or more consecutive repeated hospitalizations for MS, then all these hospitalizations were excluded, because they were likely to be related to programmed disease-modifying treatments and not to specific treatment for relapse; and (4) if a patient had only two repeated MS hospitalizations, then only the second hospitalization was excluded.

A random validity check of data was made on 100 hospitalizations at Saint Antoine hospital and showed that 88% were related to real relapses; others were diagnosis work-up and progressive aggravation without relapse. In the subset of Saint Antoine, the median delay between symptom onset and hospitalization was 13 days (first quartile 7 days, third quartile 30 days).

### PM Exposure and Other Variables

The patients' home addresses and the date of hospitalization were recorded using the administrative database of each hospital. PM_2.5_ concentrations (μg/m^3^) data were obtained through the high spatial resolution CHIMERE air quality model (17), an Eulerian offline chemistry-transport model, on a daily basis at a 1-km resolution, accounting for primary pollutant emissions, meteorological fields, topography, and chemical boundary conditions (17). The average weekly PM_2.5_ concentrations were estimated during each of the 6 weeks (week[0] to week[−5]) preceding admission.

Potential confounders of the relationship between PM_2.5_ and MS according to the literature consisted in temperature, influenza epidemics, pollen counts, and holiday periods. Meteorological parameters were obtained from the French meteorological service Méteo France. Data on influenza-like infection were obtained from the French Sentinel Network (18): the flu-like syndrome weekly rate was determined as the number of visits per week for influenza symptoms (fever, cough, sore throat, etc.) to general practitioner belonging to the French Sentinel Network. Pollen counts were obtained from the Réseau National de Surveillance Allergique (19). Holiday periods were defined as the two last weeks of December (Christmas holidays) and the whole months of July and August (summer holidays).

### Statistical Analysis

The main objective was to determine whether PM_2.5_ concentration was statistically associated with the risk of MS relapse using a case-crossover design. The secondary objective consisted in exploring the influence of age on the association between PM_2.5_ and MS relapse through stratification.

In the case-crossover study, each patient was his/her own control. In evaluating the impact of PM_2.5_ on MS relapse risk, the concentration was assessed by week before hospitalization, week[0] corresponding to the 7 days, ended by the hospitalization admission date. We used the natural logarithm of PM_2.5_ weekly exposure because it complied with the normality required by the analysis. The natural logarithm (Ln) of the average weekly PM_2.5_ concentrations for each of the 6 weeks (week[0] to week[−5]) preceding hospitalization was compared to the concentration during the previous week (e.g., PM_2.5_ exposure at week[0] was compared to PM_2.5_ concentration at week[−1], PM_2.5_ concentration at week[−1] was compared to PM_2.5_ concentration at week[−2], etc.) using a conditional logistic regression, adjusted for average weekly temperature, flu-like syndrome rate, pollen count, categorized as quartiles, and holiday period (“yes” or “no”).

Successively, in order to compare our findings with previous studies exploring PM_10_ exposure, we analyzed the impact of weekly PM_10_ exposure on MS relapse risk with the same methodology as that for PM_2.5_ using conditional logistic regression adjusted on the same confounders allowing to compare the impact and temporal relationship of PM_10_ and PM_2.5_ on MS relapse.

Lastly, we evaluated the influence of age on the association between PM_2.5_ and relapse. We thus evaluated the association during weeks significantly associated with relapses in different classes of age (under 30 years, 30–40 years, 40–50 years, over 50 years).

As MS treatment modifications can influence MS relapse, we did a sensitivity analysis, excluding patients who have switched MS treatments during the 6 weeks before hospitalization. This sensitivity analysis was performed for patients hospitalized at Saint-Antoine Hospital in which medical data were available in our local EDMUS (European Database on Multiple Sclerosis).

This observational study was based on data extracted from the French administrative database (Programme de Médicalisation des Sytèmes d'Information) in strict compliance with the French reference methodology established by the French National Commission on Informatics and Liberty (CNIL) (CNIL authorization for declaration 2060974, Ref MMF/CRX/AE171388), in accordance with the French law, including the GPRD (General Practice Research Database). All statistics were made on Stata 14 software.

## Results

**Figure 1:**
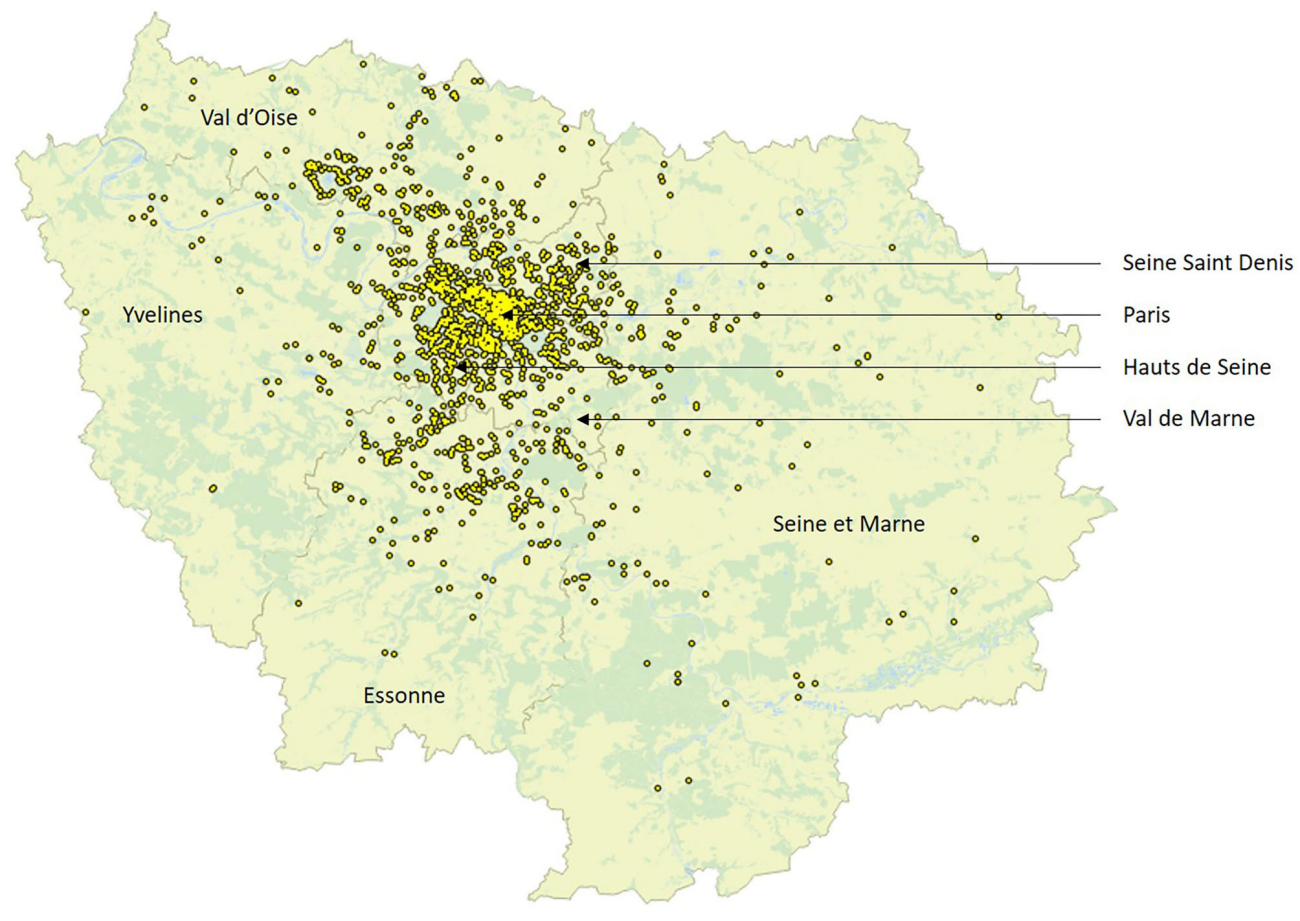
Home address of included patients in Ile de France.

A total of 2,109 hospitalizations likely due to MS relapse were included, as described in Table 1 and Figure 1. The median age of the patients was 40 years; 344, 684, 589, and 492 were, respectively, under 30, 30–40, 40–50, and older than 50 years. Thirty-one percent of the hospitalized patients were living in Paris, while the remaining patients were allocated in other departments of Ile de France.

**Table 1:** Description and characteristics of included multiple sclerosis (MS) hospitalizations.

**Total**	**2,109**
Single hospitalization	1,246
Age of patient, year, median (P25;P75)	40 (33;49)
Female, number (percentage)	1,496 (70.93)
**Department of home address**, ***N*** **(%)**	
Paris	664 (31.48)
Seine Saint Denis	296 (14.04)
Hauts de Seine	286 (13.56)
Val d'Oise	217 (10.29)
Essonne	321 (11.15)
Val de Marne	173 (8.20)
Seine et Marne	160 (7.59)
Yvelines	84 (3.98)
**Hospital**, ***N*** **(%)**	
Hôpital Pitié Salpêtrière	846 (40.11)
Fondation Rothschild	552 (26.17)
Hôpital Saint Antoine	487 (23.09)
Hôpital Pontoise	122 (5.78)
Hôpital Orsay / Longjumeau	102 (4.84)
Hospitalization duration, median (P25;P75)	3 (3;3)

The monthly distribution of the 2,109 hospitalizations revealed a peak in October (239 hospitalizations), a high rate during winter months (209 in January, 211 in February, and 209 in March), and a decreasing rate in summer (172 in June and 165 in July), with a nadir in August (85 hospitalizations). The mean PM_2.5_ concentrations of patients with addresses in Ile de France were maximal in the cold period (from October to March, mean PM_2.5_ 22.45 [confidence interval 21.79;23.11] μg/m^3^) and minimal in the warm period [from April to September, mean PM_2.5_ 14.99 (CI 14.50;15.48) μg/m^3^]. The median (25^th^; 75^th^ percentile) PM_2.5_ concentrations for week[−5] to week[0] were 15.09 (10.73;23.27), 14.88 (10.69;23.35), 15.66 (11.20;23.20), 15.76 (11.04;23.88), 15.46 (11.24;24.45), and 16.08 (11.21;24.55) μg/m^3^, respectively.

**Figure 2:**
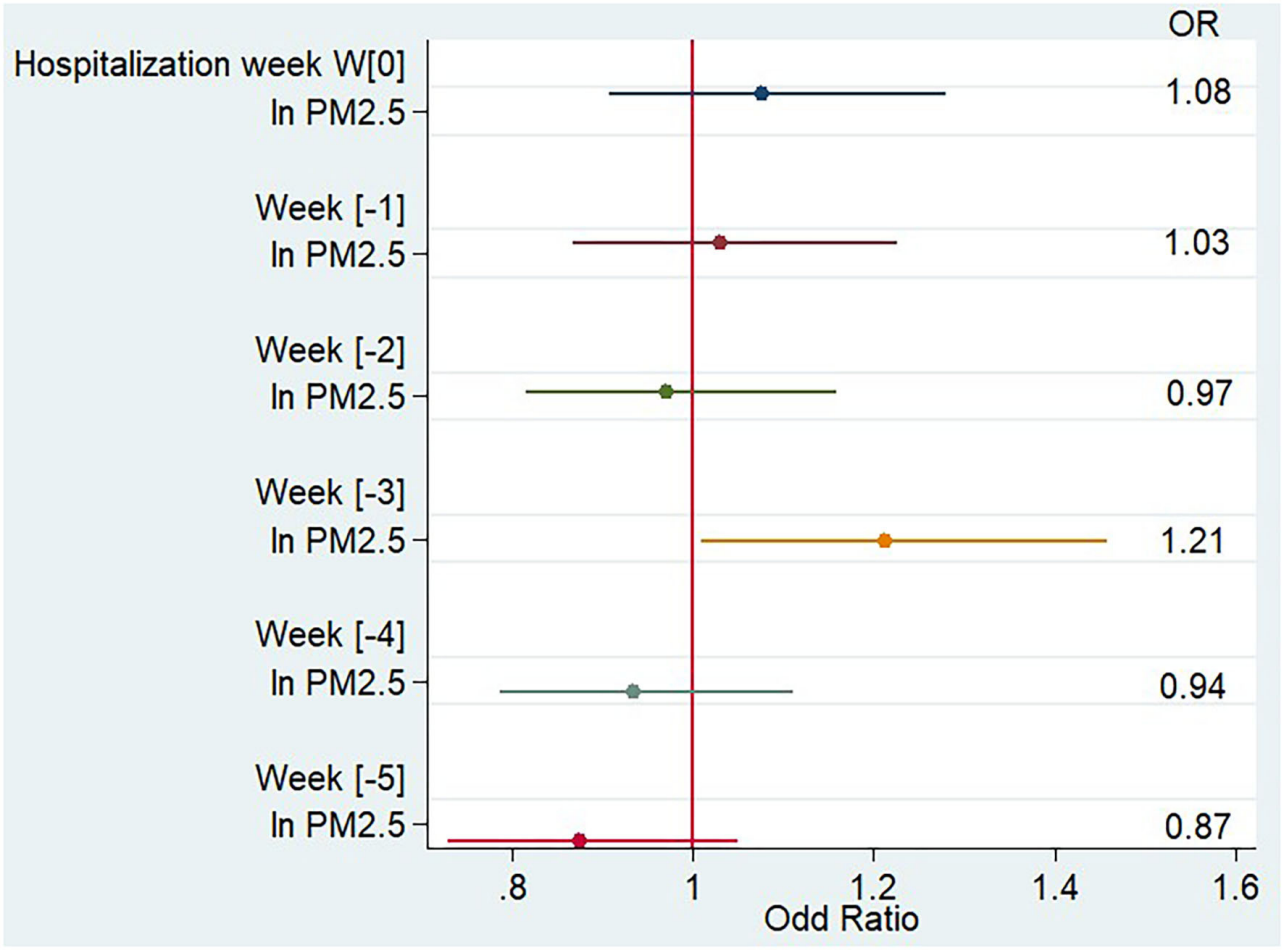
Impact of PM_2.5_ weekly concentration on the risk of multiple sclerosis relapse, multivariate analysis.

As shown in Table 2 and Figure 2, after adjustment for average weekly temperature, flu-like syndrome rate, pollen count, and holiday period, the PM_2.5_ concentration 3 weeks (week[-3]) before hospitalization (from day 21 to day 28 before hospitalization) was significantly associated with an increased risk of hospitalization likely due to MS relapse compared to PM_2.5_ concentration 4 weeks before hospitalization (odds ratio [OR] 1.21 [confidence interval {CI} 1.01;1.46]). No significant association was observed during the other weeks.

**Table 2:** Impact of PM_2.5_ weekly concentration on multiple sclerosis relapse, multivariate analysis.

**Week**	**Variable**	**Odds ratio (confidence interval 95%)**	***P*-value**
Week 0	Ln of weekly PM_2.5_	1.08 (0.91;1.29)	0.399
	Weekly mean of dairy maximal temperature (1)	0.91 (0.79;1.04)	0.158
	Weekly flu like syndrome rate (1)	0.99 (0.87;1.12)	0.825
	Weekly pollen count (1)	0.99 (0.92;1.09)	0.995
	Holliday period (2)	0.76 (0.53;1.11)	0.154
Week−1	Ln of weekly PM_2.5_	1.03 (0.88;1.23)	0.730
	Weekly mean of dairy maximal temperature (1)	0.98 (0.84;1.13)	0.801
	Weekly flu like syndrome rate (1)	0.91 (0.80;1.04)	0.158
	Weekly pollen count (1)	0.95 (0.86;1.05)	0.354
	Holliday period (2)	0.39 (0.25;0.60)	**0.001**
Week−2	Ln of weekly PM_2.5_	0.97 (0.81;1.16)	0.748
	Weekly mean of dairy maximal temperature (1)	0.83 (0.72;0.95)	**0.009**
	Weekly flu like syndrome rate (1)	1.07 (0.94;1.23)	0.297
	Weekly pollen count (1)	0.91 (0.82;0.99)	**0.036**
	Holliday period (2)	0.75 (0.51;1.10)	0.136
Week-3	Ln of weekly PM_2.5_	1.21 (1.01;1.46)	**0.039**
	Weekly mean of dairy maximal temperature (1)	0.98 (0.86;1.13)	0.798
	Weekly flu like syndrome rate (1)	1.05 (0.92;1.20)	0.457
	Weekly pollen count (1)	0.94 (0.86;1.03)	0.189
	Holliday period (2)	0.82 (0.60;1.13)	0.227
Week-4	Ln of weekly PM_2.5_	0.93 (0.79;1.11)	0.443
	Weekly mean of dairy maximal temperature (1)	0.99 (0.86;1.15)	0.938
	Weekly flu like syndrome rate (1)	1.01 (0.88;1.15)	0.492
	Weekly pollen count (1)	0.99 (0.90;1.08)	0.739
	Holliday period (2)	0.92 (0.64;1.34)	0.652
Week-5	Ln of weekly PM_2.5_	0.87 (0.73;1.05)	0.150
	Weekly mean of dairy maximal temperature (1)	0.87 (0.75;1.00)	0.054
	Weekly flu like syndrome rate (1)	0.86 (0.75;0.99)	**0.034**
	Weekly pollen count (1)	0.99 (0.91;1.09)	0.868
	Holliday period (2)	0.72 (0.51;1.00)	0.055

*(1) By increase of one quartile*.

*(2) Holidays: Christmas and summer holidays*.

*Bold values indicate p < 0.05*.

The impact of PM_10_ exposure on MS relapse risk was equivalent to the one observed for PM_2.5_ exposure, with a significant association only observed in week[−3] [OR 1.29 (CI 1.02;1.62)].

**Figure 3:**
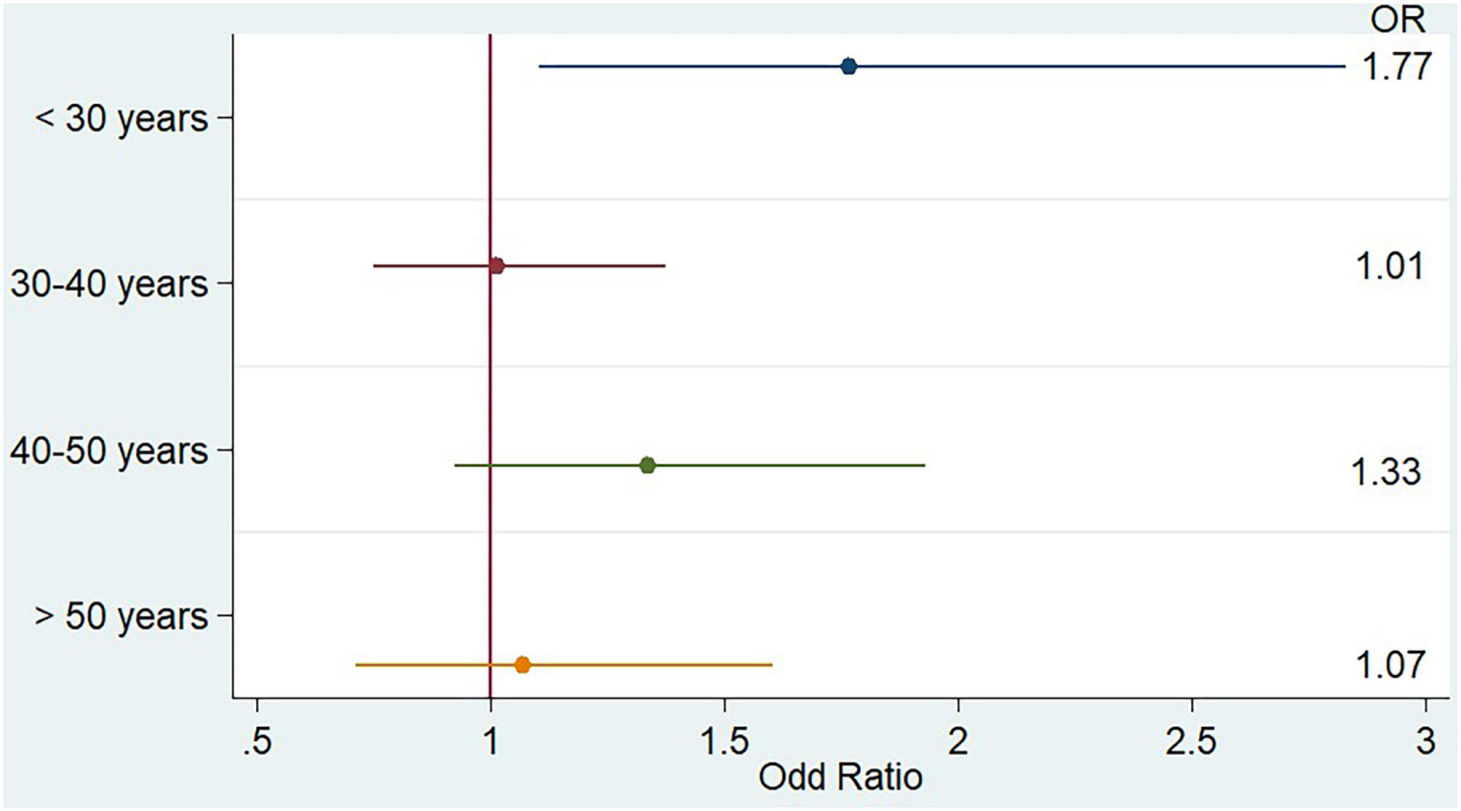
Impact of PM_2.5_ weekly concentration during week[-3] on the risk of multiple sclerosis relapse stratified by age, multivariate analysis.

When we looked at the PM_2.5_ effect by class of age, as shown in Figure 3, we noticed that PM_2.5_ association with relapse was greater and only significant in younger patients, as the OR for the 344 hospitalizations of patients younger than 30 years was 1.77 (CI 1.10;2.83). When we restricted the analysis to patients younger than 25 years during week[−3], the OR for MS relapse increased to 3.10 (CI 1.27;7.57).

A sensitivity analysis was made on patients hospitalized at Saint Antoine Hospital, of which 373 concerned patients included in our local EDMUS. Seventeen (4.5%) of these concerned patients who have initiated a new treatment during the 6 weeks before the hospitalization and were excluded. We performed the same analysis on the remaining 356 hospitalizations. PM_2.5_ remained associated with MS during week[-3] [OR 1.68 (CI 1.06; 2.66), *p* = 0.025].

## Discussion

In this retrospective study including 2,109 hospitalizations likely due to MS relapse, PM_2.5_ concentration was associated with hospitalizations, and the association was stronger in patients younger than 30 years.

We found a specific temporal relationship between PM_2.5_ concentration and hospitalization likely due to relapses for the week[-3] preceding hospitalization, suggesting that several weeks may be required following PM_2.5_ exposure for the immune response to be orchestrated and generate new lesions underlying relapses. In the subset made in Saint Antoine hospital where the relapses were verified, the first symptoms related to the relapse appeared 13 days before hospitalization, highlighting a delay between the first symptoms onset and hospitalization. This means that finally the delay between pollution peak and relapses may be shorter than 3 weeks. As MS lesions can take several days or weeks to form, first involving activation of the peripheral immune system, subsequently inducing microglial activation, oxidative stress, blood-brain barrier disruption, and demyelination (20), PM_2.5_ exposure may contribute to either the early immune system activation or to later stages of MS lesion formation, preceding clinical relapse appearance. Interestingly, a short-term impact of PM_10_ was suggested by two French studies (14, 21). In the former study, MS relapses were associated with PM_10_ during the cold season, the control days having been chosen to be ±35 days relative to the case (relapse) day. The latter study confirmed the first results but through a multipollutant analysis that also indicated ozone exposure during days 1–3 before the relapse symptom onset as a risk factor during the hot season (21).

In our study, both PM_2.5_ and PM_10_ exposures were associated with a similar increase in MS relapse risk during week[−3]. As PM_2.5_ is part of PM_10_, this result is not surprising and suggests that most of the effect may be mediated by PM_2.5_. A multivariate analysis including both PM_2.5_ and PM_10_ could not be performed due to the collinearity of both variables that are closely related in the air quality CHIMERE model.

So far, the role of PM_2.5_ has only been explored in the case of MS incidence and prevalence but not of relapse, with conflicting results. A strong positive correlation between PM_2.5_ exposure and MS prevalence was found in the province of Padova (9, 10), contrasting an absence of relation in studies having considered MS incidence in adults (22–24). However, in a case–control study of pediatric MS, worsening air quality significantly impacted the odds for MS (OR = 2.83; 95%CI 1.5, 5.4 for patients living <20 miles from a referral center, and OR = 1.61; 95%CI 1.2, 2.3 for those who resided ≥20 miles from a referral center).

Previous pathophysiological studies have supported a key role played by PM_2.5_ that would predominantly involve a proinflammatory effect. Unlike PM_10_, PM_2.5_ can reach pulmonary alveoli, where they are incorporated by alveolar macrophages, promoting macrophage production of inflammatory cytokines such as interleukin-12 and subsequently pushing T lymphocyte differentiation into Th1 profile (25) and systemic inflammation (26). Part of T lymphocyte activation may therefore be triggered in the lung, followed by a migration step toward the CNS. This sequence of events has been described as the “brain–lung axis” (27), which is considered as one of the main explanations of the negative impact of smoking on MS (28). Supporting this hypothesis, the systemic inflammation induced by carbon nanoparticles in rodents could only be observed when particles were administered by inhalation and not when they were administered by intra-arterial infusion (29). More recently, Andrea Cortese and colleagues highlighted in MS patients that PM_10_ exposure 15 days before blood sampling was associated with the production of Th-17 polarizing interleukin from monocyte derived dendritic cell and an overexpression of CCR-6 CD4+ T circulating cells, which may facilitate their entry to the CNS (30). The time for systemic immune response to be orchestrated and generate posterior damage in the CNS following the “brain–lung axis” could therefore explain the observed delay between PM_2.5_ exposure and relapse onset.

Systemic and cerebral oxidative stress are also enhanced by particulate matter exposure (31, 32), inducing mitochondrial dysfunction, involved in the initiation of MS lesion formation (“prephagocytic” lesions) (33). Therefore, oxidative stress and mitochondrial dysfunction could be another mechanism of brain damage related to PM exposure, acting synergistically with brain inflammation.

A “direct” toxic effect of PM_2.5_ on the nervous system may also be hypothesized and contribute to MS relapses, but would imply that part of the PM can reach the brain. In rodents, only PM_0.1_ can reach the brain essentially through the olfactive nerve, as shown by Elder et al. (5). An alternative way, involving a translocation of PM from the lung to systemic circulation and to the CNS, is still debated, and it has been estimated that ~13% of inhaled nanoparticles are able to translocate from the lung to the blood (34). Such a translocation from the blood to the CNS is considered poorly effective in non-MS patients (5) due to the integrity of the brain-blood barrier. However, in diseases characterized by brain–blood barrier dysfunction such as MS, this way could be more relevant, as highlighted in the EAE model, where intravenously injected nanoparticles incorporated to macrophages and neutrophils were found in the cerebral white matter (4).

In humans, a relationship between air pollution exposure and cerebral inflammation has been previously suggested in a Mexican study comparing the level of inflammatory biomarkers in the CSF of children living in a highly polluted area compared with others living in a less polluted city (35). This study showed that macrophage inhibitory factors interleukin 2 and 6 were significantly increased in the CSF of children living in a more polluted area.

Young people were particularly impacted by PM_2.5_ concentration in our study, although the confidence interval of the observed association was broad (CI 1.06; 2.66), probably because of the relative small number of hospitalized patients younger than 30 years (*N* = 344). This result may be explained in two ways. First, young age is associated with a more inflammatory and relapsing disease and with a more frequent breakdown of the blood-brain barrier (36), so that young patients may be more sensitive to environmental aggression. For other neurological diseases such as stroke, age was also found to be negatively associated with the impact of air pollution exposure (37). Second, employment is extremely rapidly declining in MS patients: in a study in Sweden, 23.7% of patients under 34 years received a disability pension, but this ratio climbed to 48.9% for those aged from 35 to 44 years and near 70% for patients over 44 years (38). Accordingly, in a longitudinal study in France, only 54.4% of MS patients were still working 7 years after MS diagnosis (39). Therefore, young people may be more likely to remain active on the labor market. Working in Ile de France often involves a long journey from home to work, and thus an increase in exposure to air pollution, which could explain the strength of the observed association in young people. In the study of Roux et al. (14) in the Strasbourg Area, there was also a trend for an increase in the PM_10_ concentration association with MS relapse for younger people [the OR for patients under 30 years was 1.48 (CI 0.91; 2.40) and for 30–40 years was 1.65 (CI 1.02; 2.68), while the OR for those older than 40 years was only 1.23 (0.82; 1.84)].

This study has some limitations. Due to the retrospective administrative database collection of data, we cannot assume that all the hospitalizations were linked to MS relapse. However, in our verification sample subset in Saint Antoine Hospital, we found nearly 90% of “true relapses” in the included hospitalizations. Second, the day hospital recruitment that has been chosen may miss part of the hospitalizations for both severe relapses (that required complete hospitalization) and mild relapses (that did not require day hospitalization for methylprednisolone). We could not stratify the analysis based on MS treatment, smoking status, or vitamin D level, but the case-crossover design allowed us to take into account these interindividual differences because each patient was his/her own control. Adjustment on influenza infection was based on ecological measures because we did not have individual data on this variable. Moreover, the time from symptom onset to hospitalization date was variable (median 13 days but first quartile 7 days, and third quartile 30 days in the verification subset of Saint-Antoine Hospital), inducing a temporal dispersion of the period of interest. However, this temporal dispersion should have attenuated the observed relationship, which remained significant in our study. Of note, air pollution was estimated at the address of included patients, and a substantial number of included patients could have passed the weeks before hospitalization elsewhere. Finally, this method of estimation does not account for the exposure during daily journeys and exposure to indoor air pollution.

Despite the mentioned limitations, our study found an association between PM_2.5_ concentration and the risk of MS relapse. Through technical improvements in the collection and analysis of air pollution data, future studies should investigate the specific effect of each component of fine particle and include ambulatory measurement in order to guide long-term measures to address the issue of air quality.

## Data Availability Statement

The raw data supporting the conclusions of this article will be made available by the authors, without undue reservation.

## Ethics Statement

Ethical review and approval was not required for the study on human participants in accordance with the local legislation and institutional requirements. Written informed consent for participation was not required for this study in accordance with the national legislation and the institutional requirements.

## Author Contributions

EJ, BS and IA-M were responsible for study conception, design, supervision, interpretation of data, and drafting of the manuscript. EJ was responsible for statistical analysis. BD was responsible for air pollution data management. AC was responsible for air pollution data. All authors contributed to the article and approved the submitted version.

## Conflict of Interest

EJ reports reimbursement for conference registration fees, travel expenses, and accommodation from Sanofi Genzyme, outside the submitted work. BS has received fees for advisory boards and lectures from Genzyme, Merck-Serono, Novartis, Teva, and Biogen, and research support from Roche, Genzyme, and Merck-Serono. The remaining authors declare that the research was conducted in the absence of any commercial or financial relationships that could be construed as a potential conflict of interest.
